# Enhancing evidence-based diabetes and chronic disease control among local health departments: a multi-phase dissemination study with a stepped-wedge cluster randomized trial component

**DOI:** 10.1186/s13012-017-0650-4

**Published:** 2017-10-18

**Authors:** Renee G. Parks, Rachel G. Tabak, Peg Allen, Elizabeth A. Baker, Katherine A. Stamatakis, Allison R. Poehler, Yan Yan, Marshall H. Chin, Jenine K. Harris, Maureen Dobbins, Ross C. Brownson

**Affiliations:** 1 Prevention Research Center in St. Louis, Brown School, Washington University in St. Louis, One Brookings Drive, Campus Box 1196, St. Louis, MO 63130 USA; 2 Department of Behavioral Science & Health Education, College for Public Health & Social Justice, Saint Louis University, St. Louis, USA; 3 Department of Epidemiology, College for Public Health & Social Justice, Saint Louis University, St. Louis, USA; 4 Department of Surgery (Division of Public Health Sciences) and Alvin J. Siteman Cancer Center, Washington University School of Medicine, Washington University in St. Louis, St. Louis, USA; 5 Department of Medicine and Chicago Center for Diabetes Translation Research, University of Chicago, Chicago, USA; 6 Brown School, Washington University in St. Louis, St. Louis, USA; 7 National Collaborating Centre for Methods and Tools and Health Evidence, McMaster University, Hamilton, Ontario Canada

**Keywords:** Dissemination research, Evidence-based public health, Public health workforce, Diabetes control, Chronic disease prevention

## Abstract

**Background:**

The rates of diabetes and prediabetes in the USA are growing, significantly impacting the quality and length of life of those diagnosed and financially burdening society. Premature death and disability can be prevented through implementation of evidence-based programs and policies (EBPPs). Local health departments (LHDs) are uniquely positioned to implement diabetes control EBPPs because of their knowledge of, and focus on, community-level needs, contexts, and resources. There is a significant gap, however, between known diabetes control EBPPs and actual diabetes control activities conducted by LHDs. The purpose of this study is to determine how best to support the use of evidence-based public health for diabetes (and related chronic diseases) control among local-level public health practitioners.

**Methods/design:**

This paper describes the methods for a two-phase study with a stepped-wedge cluster randomized trial that will evaluate dissemination strategies to increase the uptake of public health knowledge and EBPPs for diabetes control among LHDs. Phase 1 includes development of measures to assess practitioner views on and organizational supports for evidence-based public health, data collection using a national online survey of LHD chronic disease practitioners, and a needs assessment of factors influencing the uptake of diabetes control EBPPs among LHDs within one state in the USA. Phase 2 involves conducting a stepped-wedge cluster randomized trial to assess effectiveness of dissemination strategies with local-level practitioners at LHDs to enhance capacity and organizational support for evidence-based diabetes prevention and control. Twelve LHDs will be selected and randomly assigned to one of the three groups that cross over from usual practice to receive the intervention (dissemination) strategies at 8-month intervals; the intervention duration for groups ranges from 8 to 24 months. Intervention (dissemination) strategies may include multi-day in-person workshops, electronic information exchange methods, technical assistance through a knowledge broker, and organizational changes to support evidence-based public health approaches. Evaluation methods comprise surveys at baseline and the three crossover time points, abstraction of local-level diabetes and chronic disease control program plans and progress reports, and social network analysis to understand the relationships and contextual issues that influence EBPP adoption.

**Trial registration:**

ClinicalTrial.gov, NCT03211832

**Electronic supplementary material:**

The online version of this article (10.1186/s13012-017-0650-4) contains supplementary material, which is available to authorized users.

## Background

With more than 30 million people in the USA diagnosed with diabetes and 84.1 million with prediabetes [1], the burden on individuals, families, communities, and society is substantial. Racial, ethnic minority and lower socioeconomic groups bear a disproportionate burden with higher rates of prevalence and complications [2]. In addition to the health impact, medical spending per person in the USA due to diabetes doubled between 1987 and 2011 [3]. The health and financial impact call attention to the greater need for and potential economic return of diabetes control efforts [4]. Diabetes control encompasses primary prevention, i.e., promotion of physical activity, healthy eating, obesity prevention, secondary prevention, i.e., early detection, self-management. Obesity, unhealthy eating, and physical inactivity account for more than half of new diabetes cases [5, 6]. If addressed, this could have a profound effect on health disparities, incidence of diabetes as well as cardiovascular diseases, some cancers, and other chronic conditions, and quality and length of life of those diagnosed [7–10].

Practitioners in the 2800 US local health departments (LHDs) are ideally positioned to affect programs and policies related to diabetes control because of their knowledge of, and focus on, community-level needs, contexts, and resources as well as their key role interfacing with health care providers [11, 12]. These attributes allow for LHDs to assess a public health problem, adapt an appropriate program or policy, cultivate partnerships, and assure research-tested interventions—or evidence-based programs and policies (EBPPs) are effectively delivered and implemented [13–16]. LHDs are playing an increasing role in diabetes control through activities such as obesity prevention [17, 18], diabetes self-management [19, 20], and development of registries to monitor diabetes at a population-level and intervene in a timely and targeted manner [21]. A pilot study of LHDs in Missouri assessed the use of 20 diabetes-related EBPPs, feasibility of EBPPs, and personal/organizational barriers to evidence-based practice [22].

Research has demonstrated that simply making EBPPs available for adoption is not sufficient to assure their widespread use [23–25]. Many local public health practitioners are aware of EBPPs, but lack the needed skills to adapt and use them or face organizational barriers to their use [26–30]. To improve practice, LHD professionals need evidence-based information to make decisions on how to improve public health system performance and health outcomes [31]. Evidence-based public health is the process of integrating science-based interventions with community preferences to improve the health of populations [31–34]. A key aspect of evidence-based public health is evidence-based decision-making (EBDM), which involves making decisions based on the best available scientific or rigorous program evaluation evidence, applying program planning and quality improvement frameworks, engaging the community in assessment and decision-making, adapting and implementing EBPPs for specific populations or settings, and conducting sound evaluation [30, 33, 35, 36]. In support of EBDM, there are numerous analytic tools now available to increase the use of evidence-based diabetes-related interventions (e.g., the Community Guide, What Works for Health) [37, 38].

The gap between research and practice underscores the need to understand barriers and facilitators to dissemination and uptake of evidence into practice. In a study from Brownson and colleagues in two US states, participants identified communication with policymakers, use of economic evaluation, and translation of research to practice as top competency gaps for evidence-based public health [39]. Lack of incentives, inadequate relationships between people involved in research and practice, and absence of cultural and managerial support and supportive organizational climates are among the most commonly cited barriers [30, 32, 40–45]. Studies have found a strong link between the perception of organizational priority for evidence-based practices and use of research to inform program adoption and implementation [29, 41]. Relationships among health departments can facilitate dissemination of program and policies, including diabetes EBPPs [46–48]. In addition, relationships among staff within health departments and between health departments and local stakeholders are essential to successful implementation of public health programs [49, 50].

Thus, to enhance adoption of evidence-based approaches, active and multi-modal strategies are needed to build local-level public health workforce capacity and organizational support to plan, implement, evaluate, and spread EBPPs for diabetes control. Recent work to increase the use of EBPPs for chronic disease prevention among state health department practitioners utilized multiple active dissemination strategies, including multi-day in-person evidence-based decision-making workshops, supplemental webinars, and strategies to ingrain EBDM into agency processes [51, 52].

The goals of this multi-phase study are to determine effective ways to increase awareness and skills of local-level public health practitioners to apply EBDM practices and EBPPs for diabetes and other chronic disease control, increase LHD agency and employee-level use of EBDM practices to control diabetes and chronic diseases in Missouri, and increase agency level sharing of effective approaches and EBPPs with partnering organizations.

## Methods/design

### Study design

This multi-phase dissemination study funded by the National Institute of Diabetes and Digestive and Kidney Diseases (NIDDK) evaluates the adoption and application of EBDM practices and EBPPs for diabetes control among LHDs. The study is guided by a transdisciplinary team working in dissemination and implementation research, evidence-based diabetes control, and collaborative research with LHDs as well as partners serving LHDs and local-level public health practitioners. This study involves two complementary, overlapping phases. Phase 1 includes development of a survey to collect self-report data, refinement of record review and abstraction methods to collect objective data, and a local-level needs assessment that includes qualitatively assessing agency and broader system factors that facilitate or hinder dissemination of EBPPs for diabetes and chronic disease control in LHDs in Missouri. In addition, phase 1 involves collection of self-report data from a national sample of LHD practitioners working in chronic disease control. Phase 2 is a stepped-wedge cluster randomized controlled study designed to assess effectiveness of the identified dissemination strategies on local-level practitioners working in diabetes and chronic disease control at 12 LHDs in Missouri. This involves the selection of 12 LHDs in Missouri that are randomized into one of three groups that receive the intervention, i.e., dissemination strategies, for varying durations ranging from 8 to 24 months. Dissemination strategies may include training in evidence-based public health, technical assistance through a knowledge broker, providing targeted messages/emails with a short summary of the research and actions that might be taken based on the evidence, and organizational changes to assist agency leadership in prioritizing, incentivizing, enhancing capacity for, and incorporating the use of EBPPs. Study collaborators include the National Association of County and City Health Officials (NACCHO) and the Missouri Association of Local Public Health Agencies (MoALPHA). The study was approved by the institutional review board of Washington University in St. Louis. Some parts of phase 1 have been completed, and phase two planning is underway. Phase 2 is registered as a stepped-wedge cluster randomized trial (clinicaltrials.gov NCT03211832).

**Fig. 1:**
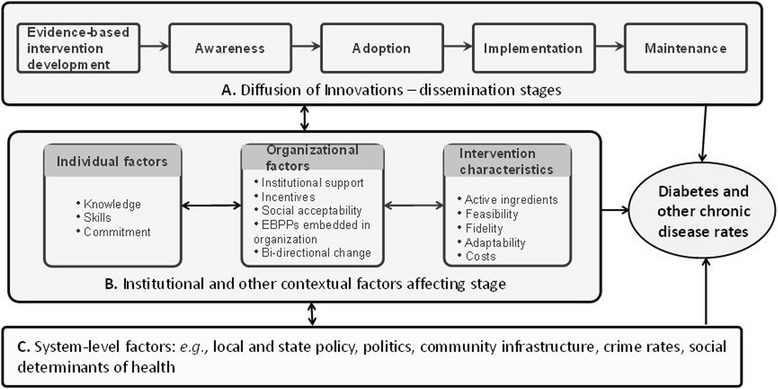
Conceptual framework of dissemination of EBPPs in LHDs

The theoretical underpinning for the study is the Diffusion of Innovation Theory [53], augmented by Institutional Theory [54–56] as depicted in Fig. 1. In the context of this study, EBPPs are the innovations, i.e., the idea or practice that is perceived as new. The decision to adopt, accept, and use an innovation is a process, and each stage of the framework has specific characteristics (see Additional file 1). An important aspect of this study’s framework is the contextual factors affecting dissemination (Fig. 1b). Individual factors can reflect the skills, knowledge, leadership, or commitment of the individuals involved in dissemination. Institutional theory draws upon a wide range of disciplines (e.g., economics, political science) to understand how social, governmental, political, and commercial institutions influence decision-making [57]. This theory explains that it is not only how individuals make decisions that influence outcomes, but also the rules that govern behavior, including formal explicit rules such as regulations and laws, and implicit rules like social norms and conventions, and self-imposed codes of conduct [58]. Key concepts from Institutional Theory that have contributed to the conceptual framework, activities, and measures are organizational support for EBPPs, incentives to utilize EBPPs, social acceptability of EBPPs, and bi-directionality of organizational change, i.e., the way organizations influence employees and vice versa [58, 59]. In addition, intervention characteristics, including feasibility, adaptability, and costs, are hypothesized to influence dissemination stage. These factors will be addressed in the menu of intervention strategies and evaluation plan.

The study team will work in partnership with local-level employees to assess contextual and organizational factors that influence adoption and use of EBPPs, and jointly plan the menu and implementation of interventions. Evaluation will include survey measures taken at pre-intervention, i.e., baseline, and at the three 8-month intervals during the study intervention period, and process evaluation in LHDs during the intervention period.

### Study audience

The audience is LHD practitioners working in diabetes and chronic disease control, including diabetes prevention and management, obesity prevention, physical activity, nutrition, cardiovascular health, and cancer screening, and their supervisors and agency leaders. The audience will be drawn from diverse backgrounds including health educators, epidemiologists, and community health nurses. Previous research has shown larger LHDs have staff who specialize in diabetes control whereas smaller LHDs typically employ more general chronic disease staff [60]. For measures development in phase 1, the study audience is LHD chronic disease control practitioners and supervisors from across the USA. For phase 2, the audience is local-level practitioners in Missouri LHDs. We will also focus on key partners for diabetes and chronic disease control in the local community, including non-governmental agencies (e.g., local hospitals, community-based organizations) and governmental agencies outside of the health sector (e.g., universities, parks and recreation, schools). Phase 2 will include a primary and secondary group in each LHD. The primary group will be made up of individuals who attend the initial intervention strategy, a targeted multi-day workshop in evidence-based public health. The secondary group will include other LHD staffs who support chronic disease control and staff from key partner agencies because they are expected to apply EBPPs for chronic diseases control as guided by the LHD and to meet sample size requirements.

### Phase 1: development of measures, local-level needs assessment

In phase 1, the study team will develop and test a survey instrument. Self-report survey data will be collected nationally, and the study team will refine a tool to abstract local health department plans and progress reports as objective data of planning and implementation of EBPPs in diabetes and chronic disease control.

### Survey instrument

The measures to assess the dissemination of EBPPs for chronic disease prevention are under-developed [61, 62] particularly among LHDs [63–65]. The primary objective is to develop a survey tool to collect key measures in phase 2. To improve the external validity of the measures [66], the survey tool will be developed at the national level. This will also provide a national picture of local-level practitioner views on EBPPs and EBDM, implementation of EBPPs among LHDs, training and informational needs for EBDM, organizational support for EBDM, barriers to EBDM, and local-level relationships including the characteristics of academic partnerships.

### Measures

The survey was developed from previous research conducted by the project team [30, 39, 51, 67–70], abstracting data from existing instruments identified through snowball sampling of the study team, and three rounds of input from November 2016–January 2017. The survey has nine sections and 86 items and was designed to be completed in 20 min. Table 1 displays the survey sections, types of items, and their sources. Based on survey data, four dependent variables will be created to tie directly with the dissemination stage within the study’s conceptual framework (Fig. 1a), i.e., awareness, adoption, implementation, and maintenance. The survey includes the Short Grit Scale, which measures passion and perseverance for long-term goals [71]. The construct of grit has been identified as a factor related to overall performance in several different populations [71], and recent research found grit to be positively associated with implementing and sustaining evidence-based interventions among front-line therapists treating substance abuse disorders [72]. The study team developed two items on health equity in programs, policies, and services, which were included in the section, Views on EBPPs. The survey instrument was programmed in Qualtrics online survey software and went through several iterations of review and refinement with research staff before cognitive response and reliability testing.

**Table 1:** Survey measures

Survey section	Number of items	Types of variables	Sample items	Item sources
Background	9	Check one, years, check all that apply, yes/no	Position, years in position, degree/credentials, gender, race/ethnicity, age, program areas	Jacobs 2010
EBPPs to address chronic diseases (selection was based on program area)	8	Yes/no/do not know	Asked 1 topic: diabetes control, obesity prevention, physical activity, nutrition, or tobacco control Has your agency directly delivered? (4 items) Has your agency collaborated with organization(s) to support delivery? (4 items)	Community guide, What works for health, Guide to clinical preventive services
Views on EBPPs	6	Likert 7-point scale, text/short answer	Program staff in my work group/division is aware of toolkits for planning and evaluation. Health equity (2 items)	Jacobs 2010, Reis 2014 New
EBDM definitions and supports	2	Rank top 3	Which of the following would be most useful to you in building skills for EBDM?	Jacobs 2012, Reis 2014
Importance and availability of EBDM skills	20	Likert 11-point scale	Importance (10 items) Availability (10 items)	Brownson 2009
Spreading EBDM	26	Likert 7-point scale	Dissemination stages (22 items) Administrative evidence-based practices (4 items)	Stamatakis 2017, Brownson 2012, 2013
Ending programs	4	Select top 3, how often	Reasons for programs ending that should have continued, reasons for continuing programs that should have ended	Brownson 2015
Work style	8	Likert 5-point scale	Short Grit Scale	Duckworth 2009
Academic partnerships	2	Yes/no/unsure, check all that apply	Does your agency currently participate in any academic partnerships?	New

### Survey instrument testing

#### Cognitive response testing

Survey items were revised through cognitive response testing, which has been shown to improve survey development and the quality of data collection [73]. Ten local public health practitioners working in diabetes and chronic disease control at LHDs across the USA identified by a partner organization completed hour-long phone interviews in February and March 2017. Interviews were conducted by the project manager and involved reviewing survey items with participants. Participants provided feedback on question comprehension (what the participant thought the question was asking, question wording clarity) and information retrieval and decision processing (questions that were clear but difficult to answer). In addition, participants shared input on the relevance of EBPPs to local-level practitioners and LHDs. Interview participants were offered a $20 Amazon.com gift card for completion of the cognitive response testing. Interviews were audio-recorded and reviewed to identify themes that occurred in two or more interviews. A summary report was reviewed by the study advisory group who refined item wording.

### Reliability test-retest

A list of 100 LHDs was randomly selected from LHDs who completed the 2016 NACCHO National Profile and reported that their LHD screens for diabetes or body mass index (BMI) or conducts population-based primary prevention activities for nutrition or physical activity. The lead chronic disease control staff person was identified for each LHD selected. Respondents that completed the survey the first time were emailed an invitation to take the survey again 14–21 days after their initial survey. Efforts were made to distribute the sample across LHD jurisdiction population size (small < 50,000, medium 50,000–199,999, and large ≥ 200,000). Replacement sampling was done as needed to get close to 100 eligible invitees. Of the 97 eligible practitioners invited, 56 completed test 1 (57.7% response rate), and 53 completed test 2 (94.6% of test 1). Respondents completed the second survey 15 to 36 days after the first survey.

Statistical analyses for test-retest included calculating intra-class correlation coefficients (ICC) for Likert scale items and percent agreement and Cohen’s kappa statistic for dichotomous items and dichotomized Likert-scale items (strongly agree and agree vs. other responses). Cronbach’s alpha was calculated to test internal consistency of the domain and influence of individual items on a domain for each continuous variable. For ranking items, the percent agreement of the three items chosen in the top three in test 1 and test 2 was calculated. Landis and Koch [74] kappa categories of almost perfect (1.0–0.8), substantial (0.8–0.6), moderate (0.6–0.4), fair (0.4–0.2), and low (0.2–0.0) were used to interpret the results. For ICCs and percent agreement, > 0.60 was considered desirable and > 0.70 was good, respectively [75]. Test-retest results showed that percent agreements were typically ≥ .70 and ICCs, the best fitting statistic for most sections and items, were mostly ≥ .60. The majority of kappa coefficients were in the moderate and substantial ranges (0.4–0.6 and 0.6–0.8). Most of the scales showed good internal consistency (Cronbach’s alpha > .80). Based on the review of test-retest results, the wording of eight items was revised, and one item was split into two. Since the survey was only slightly modified, test 1 surveys will be combined with the full survey sample data described below for almost all items.

### Survey participant recruitment

**Fig. 2:**
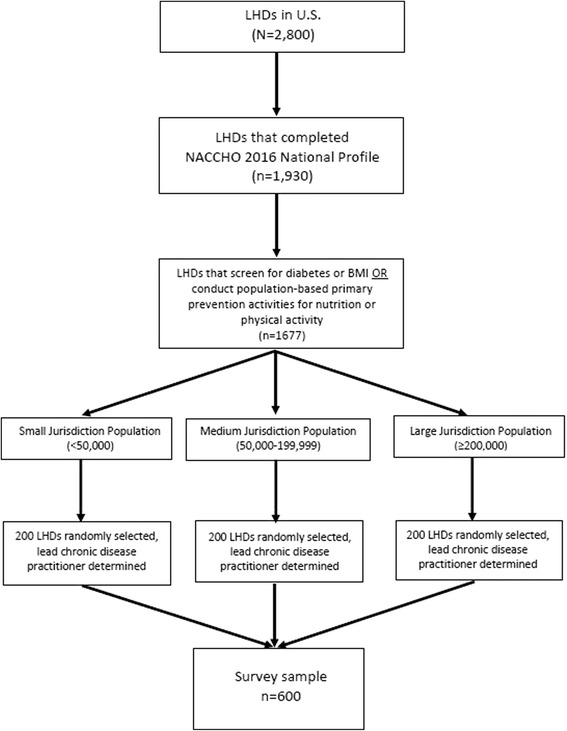
Phase 1 national survey selection of LHD lead practitioners working in chronic disease control

Figure 2 depicts the selection of LHD lead practitioners working in chronic disease control for the national survey, similar to reliability test-retest selection mentioned previously. Efforts were made to distribute the sample across LHD jurisdiction population size. Data will be collected using Qualtrics online survey software. Pre-invitation emails, informing survey contacts about the study purpose, will be sent 1 week prior to sending the survey invitations, which will also include study information and the survey link. Follow-up to non-respondents will include three reminder emails and two follow-up calls. Respondents will be offered a $20 Amazon.com gift card for completing the survey. A survey response rate of 66.7% is anticipated.

### Program record review tool development

Phase 1 includes the development and testing of a tool and codebook for record abstraction of LHD plans and reports for diabetes control. The purpose is to supplement the self-reported data on EBPPs being planned and implemented for diabetes control in the twelve participating LHDs (phase 2). To draft the record abstraction tool and codebook, the study team will abstract work plans, strategic plans, and reports from 25 LHDs that participate in the national survey. The Community Guide [76], What Works for Health [38], and The Guide for Clinical Preventive Services [77] will be the basis for EBPP inclusion on the tool for program areas. Currently, there are no systematic records among LHDs in Missouri capturing diabetes prevention and management efforts; however, the study team will proceed with abstracting LHD work plans, strategic plans and progress reports in diabetes prevention and management, obesity prevention, physical activity, nutrition, and tobacco control from the selected LHDs during pre-intervention (baseline) and after dissemination strategies are applied.

### Local-level needs assessment

Since diabetes control covers an array of risk factors, demographic considerations, and intervention approaches, we conducted a needs assessment with LHDs in Missouri to narrow, prioritize, and tailor approaches to LHDs in Missouri. This will allow us to refine the menu of dissemination strategies for use in phase 2. The needs assessment process included compiling and organizing diabetes mortality, prevalence and incidence, and risk factor data for Missouri counties into tables and maps to analyze the burden of diabetes, and organizing potential interventions by diabetes risk factors. In addition, we conducted qualitative phone interviews of key informants to assess organizational, inter-organizational, and broader contextual factors that facilitate or hinder dissemination and implementation of EBPPs for diabetes and chronic disease control. Among the 115 LHDs in Missouri, we conducted 24 1-hour, key informant interviews from 14 LHDs in January–April 2017 (14 local health directors, and 10 employees/practitioners who lead diabetes or chronic disease control for their LHD). The interviews were audio-recorded, transcribed verbatim, and reviewed for completeness and accuracy. Interviews were coded by two coders using a consensus coding approach. The coding approach included segmentation of text using the interview guide to establish major categories, codebook development, coding, assessment of reliability, codebook adjustment, and final coding with iterative modifications [78–81]. Themes will be identified, and a summary report that includes a list of potential dissemination activities will be developed and shared with the study advisory committee, which will include practitioner partners and participating LHD designees. The advisory committee will be asked to rate the usefulness and feasibility of each possible dissemination activity. This rating process will allow us to tailor intervention activities to LHDs’ priorities and needs. This is likely to increase the effectiveness and sustainability of dissemination approaches.

### Phase 2: dissemination with local health departments

#### Overview

**Fig. 3:**
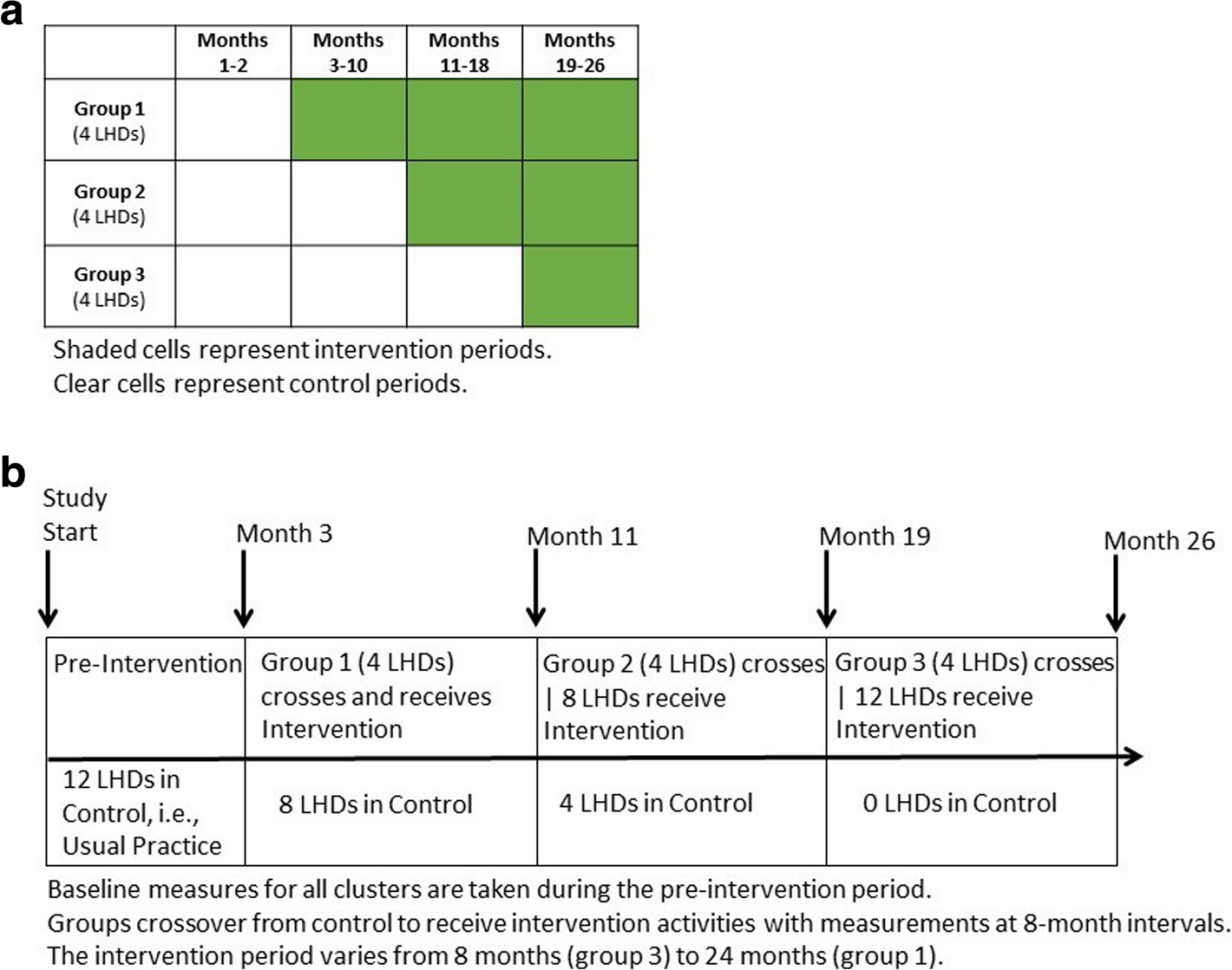
**a** Phase 2 stepped wedge study design. **b** Stepped wedge implementation during study

Phase 2 will be a stepped-wedge cluster randomized evaluation study to assess the effectiveness of active dissemination approaches on local-level practitioners to increase adoption and use of EBPPs for diabetes and chronic disease control among LHDs in Missouri. Figure 3 depicts the phase 2 stepped wedge design and implementation during the study. Unlike parallel trials, the stepped wedge design randomly assigns clusters to the order of implementation such that all clusters eventually receive the intervention [82]. This design has been primarily used to evaluate interventions during routine implementation, mainly for interventions where there is lack of evidence of effectiveness but there is a strong belief the intervention will do more good than harm [83], which was deemed the instance for this study by the study team and advisory group. The clusters are LHDs and are made up of individual employees working in or supporting diabetes or chronic disease control. We will select and recruit 12 LHDs from the 115 LHDs in Missouri. LHDs will be randomly assigned to three groups that cross over from the control, i.e., usual practice, to participate in the intervention with measurements at 8-month intervals. No LHDs receive the intervention at baseline. All participating 12 LHDs will participate in the intervention, however, for varying durations; group 1 crosses over to receive the intervention activities first, and for a total of 24 months, group 2 crosses over second (at 11 months) and receives the intervention for 16 months, and group 3 crosses over last (at 19 months) and receives the intervention activities for 8 months. The intervention activities seek to build workforce and organizational capacity and to effectively package and provide information so that it is timely, relevant, and useful for various local-level practitioners. A menu of dissemination strategies co-developed by the advisory group and study team will be offered and then selected by each LHD. Evaluation measures will include the survey and record abstraction tool developed in phase 1 and social network analyses.

### Local health department selection and recruitment

LHD selection will be based in part on the total number of full-time equivalent (FTE) employees (number of FTEs increases with size of population served by LHD [84]), number of employees working in or supporting diabetes or chronic disease control, and diabetes burden, specifically mortality rate for diabetes as underlying cause (a measure of health disparity). Of the 12 LHDs selected and recruited, at least three will come from LHDs in counties from the highest tertile of mortality rate for diabetes as underlying cause. To be eligible, LHDs are required to have ≥ 5 employees working in or supporting diabetes or related areas in chronic disease control, which includes program areas of diabetes prevention and management, obesity prevention, physical activity, nutrition, cardiovascular health, school health, and cancer screenings. In addition, each LHD must have a designee to work with the study team on aspects of the intervention. LHDs will be assigned to one of the three groups (four LHDs to each group) using simple random concealed allocation performed by the statistician (YY).

Based on our preliminary studies and values of ICC in the literature [29, 30, 85–90], we estimated a range of effect sizes and ICCs. We calculated a median ICC from similar studies and developed a range based on a 50% decrease and increase around the median (range 0.009 to 0.027). Using previous work with LHD practitioners on EBDM competency gaps, i.e., needed but unavailable skills [90], the sample size was based on testing two hypotheses: (1) the null hypothesis of a change in mean EBDM competency gap score for a single competency (action planning) from baseline in both control and intervention conditions is 0.03, and the alternative hypothesis of a change in mean competency gap score for action planning in intervention condition is 1.000 (common SD = 2.3) and (2) the null hypothesis of a change in mean EBDM competencies summary gap score from baseline in both control and intervention conditions is 0.4, and the alternative hypothesis of a change in mean summary gap score in intervention condition is 6.1 (common SD = 18.5). Following Baio et al. [91], 12 LHDs with 3 steps (or groups of 4 LHDs each) and 20 subjects in each LHD (total = 240), using the conservative estimate of ICC at 0.02 with two-sided 5% significance level, will give more than 95% of power to reject the first null hypothesis, and 80% of power to reject the second null hypothesis.

LHDs with a total number of FTEs ≥ 35 or serving a population of ≥ 200,000 are anticipated to have an adequate number of employees working in diabetes and chronic disease control. The project manager (RP) contacted directors at LHDs with 25–34 FTEs and serve a population of < 200,000 (8 LHDs) to determine if their LHD has ≥ 5 employees working in diabetes prevention and management, obesity prevention, physical activity, nutrition, cardiovascular health, or cancer screenings; 5 of the 8 LHDs met this criterion. Of eligible LHDs (17 of 115 LHDs), those with the greatest diabetes burden (i.e., in the highest tertile for mortality rate for diabetes as underlying cause) followed by LHDs with the largest total number of FTE employees will be contacted and recruited. Random assignment of the first 12 selected LHDs to groups will be performed. The principal investigator will invite local health directors from each selected LHD to have their employees working in diabetes and chronic disease control participate in the study. If any LHD declines, the next LHD according to total number of FTE employees will be selected. All employees working in or supporting diabetes and chronic disease control in participating LHDs will be invited into the study (complete listing), and a purposive sample of individuals from partner agencies (other governmental and non-governmental) in each LHD local community will also be invited. LHDs’ key partners will be recruited into the study with the help of the chronic disease managers and other practitioners. We anticipate a range of 5 to 22 participants from the LHDs, coupled with staff from partner agencies for an average of 22 per jurisdiction.

### Dissemination strategies

In each LHD, a primary group of employees working in or supporting diabetes and chronic disease control and staff from key partnering agencies will collaborate to select and refine dissemination strategies relevant to their agency’s priorities, as well as community and broader contexts. The objective is to enhance the capacity of local-level practitioners; their work group/division and overall LHD to plan, implement, evaluate, and spread EBPPs for diabetes and chronic disease control. There will be a menu of dissemination intervention activities from which LHD practitioners can choose. Potential activities include a targeted workshop in evidence-based public health, technical assistance through a knowledge broker, targeted messages, and organizational changes to create climates that are more supportive of evidence-based approaches to diabetes and chronic disease control (see Additional file 2). Additional dissemination intervention menu activities will be generated based on the local-level needs assessment described previously. This approach circumvents the disadvantages of a universal or standard process that is unlikely to be effective across the 12 participating LHDs. To reduce exposure variability within the intervention, LHDs will be asked to choose a minimum of three and maximum of four activities from the menu. This study will not attempt to evaluate a single dissemination intervention but will pursue active, multi-modal approaches, since these are supported in the literature [62, 92–94]. The initial dissemination activity for each group of LHDs will be a multi-day in-person training course [95].

### Evaluation for phase 2

To evaluate the effects of intervention strategies, key variables that relate to the dissemination stages of awareness, adoption, implementation, and maintenance (Fig. 1a) will be measured as well as a two-part adoption variable based on the record review. Primary outcomes will be measured through individual- and organization-level self-report survey items determined through phase 1 (see Table 1). Quantitative data will come from three instruments: the national survey instrument, the record abstraction tool, and a social network analysis instrument to be developed with input from the study advisory group. Survey instrument data will be collected from individual local public health practitioners during the pre-intervention period and at the three 8-month intervals/steps during the intervention period. Records for data abstraction will be obtained from LHDs, and social network analysis instrument data will be collected from individual local public health and key partner employees at pre-intervention and at the 24-month time point, i.e., the end of the intervention period.

A variety of individual-level variables will be captured from the national survey instrument, including sociodemographic characteristics (e.g., age, education level), their health promotion practices, and individual-level knowledge about EBPPs. Organizational level variables will be tied to Institutional Theory and will include the size of the agency in which the respondent works, the agency support for EBPPs (derived from the national survey instrument), and agency/administrator awareness of EBPPs. At the local level, a variety of relevant archival variables are available and may be included: LHD expenditures on chronic diseases and health behaviors from all sources, mortality rate for diabetes as underlying cause (diabetes disparity), percent of population of non-Hispanic white race/ethnicity, presence of a local board of health, and dominant local political party. Both at the individual level and organizational level, we will measure exposure to the dissemination activities to determine whether higher exposure leads to more progression across the dissemination stages. Additionally, we will track which EBPPs are being implemented, and how closely fidelity is maintained as specific EBPPs are implemented across LHDs and adapted to address disparities and other contextual factors. As phase 2 intervention strategies are implemented with LHDs, process measures will be captured, such as staff time needed to coordinate the trainings and participation rates across sites.

### Data analyses

Using the data collected from the cross-sectional phase 1 national survey, confirmatory factor analysis will be conducted to reduce and refine the dissemination stage variable groupings while examining construct validity of the measures. Descriptive statistics will be calculated for all variables. Chi-squared and *t* tests will be done to compare subgroups of participants, and multivariate linear and logistic regression modeling will be conducted to test for hypothesized associations. For phase 2, the leading statistical method is a generalized linear mixed effect regression, which has the following form: g(E(Y_ijk_)) = μ + α_i_ + γ_ik_ + β_j_ + θ × X_ij_ + λ × Z_ik_, where Y_ijk_ is the outcome measurement for subject k at step j in LHD i. g(.) is link function of expectation of Y_ijk_ to a linear predictor. For binary outcome, g(.) is logit, and for continuous outcome g(.) is identity. α_i_ is cluster-specific random effects, capturing the correlation among outcome measurements from subjects in the same LHD; γ_ik_ is subject-specific random effects, capturing the correlation among outcome measurements within same subject over time; β_j_ is the fixed effect for time j; X_ij_ is an intervention indicator (1 if LHD i in intervention condition at time j, 0 otherwise); θ is the intervention effect; Z_ik_ is a vector of subject-level covariates for adjustment; and λ is the a vector of regression coefficients for these covariates.

### Study status

The study is currently in progress. Some phase 1 activities have been completed as previously indicated, including survey cognitive response testing and test-retest reliability data collection. The local-level needs assessment, including the qualitative assessment of organization and broader system factors that facilitate or hinder dissemination of EBDM and EBPPs for diabetes control, is close to completion. The planning of phase 2, the stepped-wedge cluster randomized trial, is in progress. LHDs selection criteria have been established and recruitment is underway.

## Discussion

This study has the likelihood to be innovative in numerous ways. To our knowledge, this is the first large-scale study of its kind among LHDs in the USA that seeks to further EBDM in diabetes and chronic disease control. This study is also among the first to (1) develop, test, and use measures of dissemination and implementation of EBPPs that are currently lacking [61, 62, 93] and (2) apply constructs and measures from Institutional Theory with Diffusion of Innovations theory, a widely used theory in public health and dissemination research. This study will apply known dissemination strategies and lessons from Canada [86, 96, 97]. The use of social network analysis has high potential to explain key relationships, network structures associated with evidence diffusion (e.g., centralized versus decentralized), and network predictors of dissemination [98–100]. In addition, this study uses a stakeholder-driven, flexible approach, with selection of dissemination strategies that have the greatest feasibility among the LHDs enrolled in the phase 2 evaluation study. A project of this scale can provide a replicable model for dissemination and has the potential to begin to shift the paradigm on how research can be more effectively scaled-up across the USA to those in an ideal position to use the evidence.

The study is subject to limitations. Due to the complexity of the phase 2 evaluation study, it will be confined to LHDs in Missouri. However, Missouri is often considered a microcosm of the USA due to how closely it mirrors the USA on a number of key characteristics including urban-rural split, diabetes disparities, race, age, and education level [101]. In addition, LHDs in Missouri operate independently of each other and are independent of state and federal public health agencies like most LHDs in the USA [84]. The pool of eligible LHDs is limited to primarily large and some mid-sized LHDs to ensure adequate sample size for self-report data collection. In addition, there is a limited pool of individuals that can be recruited for self-report data collection. Another limitation to our evaluation efforts is study activities will be completed within a dynamic natural setting that includes a push for use of EBPPs by the state health department and other funders, thus making it challenging to find differences between LHDs and attributing this difference to the intervention. With data collection triangulation efforts, we increase the study’s capability to examine our multi-approach influence. An approach with LHDs presents challenges including employee turnover and funding constraints. In phase 2, employee turnover will be monitored and managed through regular communication with practitioners in the 12 LHDs. This study will seek to address funding constraints and challenges by training employees on communicating prevention and management priorities to key decision-makers, making public health evidence available in ways that save employees time to access and digest, and providing technical assistance in grant writing and diversification of funding sources.

In conclusion, a reduction in diabetes burden and disparities is possible with the application of programs and policies proven for diabetes and chronic disease control [10]. The findings from this study will further a growing knowledge base for how best to support use of evidence-based public health practice among LHDs.

## Additional files

Additional file 1:  Diffusion of Innovation Theory - Stages and Characteristics. (DOCX 38 kb)

Additional file 2: Examples of Intervention Dissemination Strategies and Description. (DOCX 22 kb)

## Abbreviations

BMI: Body mass index
EBDM: Evidence-based decision-making
EBPPs: Evidence-based programs, policies, and other interventions
FTE: Full-time equivalent
ICC: Intra-class correlation coefficients
LHDs: Local health departments
MoALPHA: Missouri Association of Local Public Health Agencies
NACCHO: National Association of County and City Health Officials
NIDDK: National Institute of Diabetes and Digestive and Kidney Diseases

## References

[1] Centers for Disease Control and Prevention (2017). National Diabetes Statistics Report, 2017 Estimates of Diabetes and Its Burden in the United States.

[2] Peek ME, Cargill A, Huang ES (2007). Diabetes Health Disparities: A Systematic Review of Health Care Interventions. Medical care research and review : MCRR..

[3] Zhuo X, Zhang P, Kahn HS, Bardenheier BH, Li R, Gregg EW. Change in Medical Spending Attributable to Diabetes: National Data From 1987 to 2011. Diabetes Care. Jan 15 2015. 10.2337/dc14-1687 25592194

[4] Zhuo X, Zhang P, Hoerger TJ. Lifetime Direct Medical Costs of Treating Type 2 Diabetes and Diabetic Complications. *American Journal of Preventive Medicine.* 9//. 2013;45(3):253–61. 10.1016/j.amepre.2013.04.017 23953350

[5] Psaltopoulou T, Ilias I, Alevizaki M (2010). The role of diet and lifestyle in primary, secondary, and tertiary diabetes prevention: a review of meta-analyses. Rev Diabet Stud..

[6] Ley SH, Ardisson Korat AV, Sun Q (2016). Contribution of the Nurses' Health Studies to Uncovering Risk Factors for Type 2 Diabetes: Diet, Lifestyle, Biomarkers, and Genetics. American journal of public health..

[7] Schulze MB, Hu FB (2005). Primary prevention of diabetes: what can be done and how much can be prevented?. Annual review of public health..

[8] Brownson RC, Haire-Joshu D, Luke DA (2006). Shaping the context of health: a review of environmental and policy approaches in the prevention of chronic diseases. Annual review of public health..

[9] Remington P, Brownson R, Wegner M (2010). *Chronic Disease Epidemiology and Control*.

[10] Remington P, Brownson R, Wegner M (2016). *Chronic Disease Epidemiology and Control*.

[11] National Association of County and City Health Officials. Diabetes. 2014; http://www.naccho.org/topics/hpdp/diabetes/.

[12] National Association of County and City Health Officials. *Local Health Departments Protect the Public’s Health.* Washington, DC: National Association of County and City Health Officials;2014.

[13] IOM (1988). Committee for the Study of the Future of Public Health. *The Future of Public Health*.

[14] Green LW, Ottoson JM, Garcia C, Hiatt RA. Diffusion theory, and knowledge dissemination, utilization, and integration in public health. *Annual review of public health.* Jan 15 2009. 10.1146/annurev.publhealth.031308.100049 19705558

[15] Institute of Medicine. *The Future of the Public's Health in the 21st Century.* Washington, D.C.: National Academies Press; 2003.

[16] Yancey AK, Fielding JE, Flores GR, Sallis JF, McCarthy WJ, Breslow L (2007). Creating a robust public health infrastructure for physical activity promotion. Am J Prev Med..

[17] Chen ZA, Roy K, Gotway Crawford CA (2012). Obesity prevention: the impact of local health departments. Health Serv Res..

[18] Stamatakis KA, Leatherdale ST, Marx CM, Yan Y, Colditz GA, Brownson RC (2012). Where is obesity prevention on the map?: distribution and predictors of local health department prevention activities in relation to county-level obesity prevalence in the United States. J Public Health Manag Pract..

[19] Hosler AS, Zeinomar N, Asare K (2011). Diabetes-related services and programs in small local public health departments, 2009-2010. Prev Chronic Dis..

[20] Zhang X, Luo H, Gregg EW (2010). Obesity prevention and diabetes screening at local health departments. American journal of public health..

[21] Steinbrook R (2006). Facing the diabetes epidemic--mandatory reporting of glycosylated hemoglobin values in New York City. N Engl J Med..

[22] Zwald M, Elliott L, Brownson RC, Skala M. Evidence-Based Diabetes Prevention and Control Programs and Policies in Local Health Departments. *Diabetes Educ.* Aug 21 2015. 10.1177/0145721715601736 PMC4794985 26297714

[23] Fisher EB, Brownson RC, Eyler AA, Haire-Joshu DL, Schootman M, Woolf SH (2001). *Interventions to Promote Key Behaviors in Cancer Prevention and Early Detection*.

[24] Glanz K (1997). Behavioral research contributions and needs in cancer prevention and control: dietary change. Prev Med..

[25] Hannon PA, Maxwell AE, Escoffery C (2013). Colorectal Cancer Control Program grantees' use of evidence-based interventions. Am J Prev Med..

[26] Lawrence RS (1990). Diffusion of the U.S. Preventive Services Task Force recommendations into practice. J Gen Intern Med..

[27] Woolf SH, DiGuiseppi CG, Atkins D, Kamerow DB (1996). Developing evidence-based clinical practice guidelines: lessons learned by the US Preventive Services Task Force. Annual review of public health..

[28] Briss PA, Brownson RC, Fielding JE, Zaza S (2004). Developing and Using the Guide to Community Preventive Services: Lessons Learned About Evidence-Based Public Health. Annual review of public health..

[29] Brownson RC, Ballew P, Dieffenderfer B (2007). Evidence-based interventions to promote physical activity: what contributes to dissemination by state health departments. Am J Prev Med..

[30] Jacobs JA, Dodson EA, Baker EA, Deshpande AD, Brownson RC (2010). Barriers to evidence-based decision making in public health: a national survey of chronic disease practitioners. Public Health Rep..

[31] Brownson RC, Fielding JE, Maylahn CM (2009). Evidence-based public health: A fundamental concept for public health practice. Annual review of public health..

[32] Baker EA, Brownson RC, Dreisinger M, McIntosh LD, Karamehic-Muratovic A (2009). Examining the role of training in evidence-based public health: a qualitative study. Health Promot Pract..

[33] Brownson RC, Gurney JG, Land G (1999). Evidence-based decision making in public health. Journal of Public Health Management and Practice..

[34] Kohatsu ND, Robinson JG, Torner JC (2004). Evidence-based public health: an evolving concept. Am J Prev Med..

[35] Brownson RC, Fielding JE, Maylahn CM. Evidence-based decision making to improve public health practice. *Front Public Health Serv Syst Res.* 2013;2.

[36] Brownson RC, Fielding JE, Maylahn CM. Evidence-based public health: a fundamental concept for public health practice. *Annual review of public health.* 2009;30. 10.1146/annurev.publhealth.031308.100134 19296775

[37] Zaza S, Briss PA, Harris KW (2005). *The Guide to Community Preventive Services: What Works to Promote Health?*.

[38] University of Wisconsin Population Health Institute. Using What Works for Health. 2016; http://www.countyhealthrankings.org/roadmaps/what-works-for-health/using-what-works-health. Accessed July 28, 2016.

[39] Jacobs JA, Clayton PF, Dove C (2012). A survey tool for measuring evidence-based decision making capacity in public health agencies. BMC Health Serv Res..

[40] Armstrong R, Waters E, Roberts H, Oliver S, Popay J (2006). The role and theoretical evolution of knowledge translation and exchange in public health. J Public Health (Oxf)..

[41] Dobbins M, Cockerill R, Barnsley J, Ciliska D (2001). Factors of the innovation, organization, environment, and individual that predict the influence five systematic reviews had on public health decisions. Int J Technol Assess Health Care..

[42] Maylahn C, Bohn C, Hammer M, Waltz E (2008). Strengthening epidemiologic competencies among local health professionals in New York: teaching evidence-based public health. Public Health Rep..

[43] Sosnowy CD, Weiss LJ, Maylahn CM, Pirani SJ, Katagiri NJ (2013). Factors affecting evidence-based decision making in local health departments. Am J Prev Med..

[44] Winterbauer NL, Bridger CM, Tucker A, Rafferty AP, Luo H (2015). Adoption of Evidence-Based Interventions in Local Health Departments: "1-2-3 Pap NC". Am J Prev Med..

[45] Jacob RR, Baker EA, Allen P (2014). Training needs and supports for evidence-based decision making among the public health workforce in the United States. BMC Health Serv Res.

[46] Harris JK (2013). Communication ties across the national network of local health departments. Am J Prev Med..

[47] Yousefi-Nooraie R, Dobbins M, Marin A (2014). Social and organizational factors affecting implementation of evidence-informed practice in a public health department in Ontario: a network modelling approach. Implementation science : IS..

[48] Hardy AK, Nevin-Woods C, Proud S, Brownson RC. Promoting Evidence-Based Decision Making in a Local Health Department, Pueblo City-County. Colorado. *Prev Chronic Dis.* 2015;12:E100. 10.5888/pcd12.140507 PMC4492218 26111156

[49] Beatty K, Harris JK, Barnes PA (2010). The role of interorganizational partnerships in health services provision among rural, suburban, and urban local health departments. J Rural Health..

[50] Merrill J, Keeling JW, Carley KM (2010). A comparative study of 11 local health department organizational networks. J Public Health Manag Pract..

[51] Allen P, Sequeira S, Jacob RR (2013). Promoting state health department evidence-based cancer and chronic disease prevention: a multi-phase dissemination study with a cluster randomized trial component. Implementation Science..

[52] Brownson R, Allen P, Jacob R, deReyter A, Lakshman M, et al. Controlling Chronic Diseases through Evidence-Based Decision Making: A Group-Randomized Trial. *Preventing Chronic Disease.* In press. 10.5888/pcd14.170326 PMC5716810 29191262

[53] Rogers EM. *Diffusion of Innovations*. Fifth ed. New York: Free Press; 2003.

[54] March J, Olsen J (1984). The new institutionalism: organizational factors in political life. American Political Science Review..

[55] North D (1990). *Institutions, Institutional Change and Economic Performance*.

[56] Scott W (2008). *Institutions and Organizations: Ideas and Interests*.

[57] Klein P (1999). *New Institutional Economics, SSRN Working Paper*.

[58] North D (1993). *New Institutional Economics and Development, Working Paper*.

[59] DiMaggio P, Powell W, Powell W, DiMaggio P (1991). The iron cage revisited: Institutional isomorphism and collective rationality. *The New Institutionalism in Organizational Analysis*.

[60] Erwin PC, Harris JK, Smith C, Leep CJ, Duggan K, Brownson RC. Evidence-Based Public Health Practice Among Program Managers in Local Public Health Departments. *J Public Health Manag Pract.* Nov 18 2013. 10.1097/PHH.0000000000000027 PMC4703040 24253406

[61] Rabin BA, Brownson RC, Kerner JF, Glasgow RE (2006). Methodologic challenges in disseminating evidence-based interventions to promote physical activity. Am J Prev Med..

[62] Rabin BA, Glasgow RE, Kerner JF, Klump MP, Brownson RC (2010). Dissemination and implementation research on community-based cancer prevention: a systematic review. Am J Prev Med..

[63] Fielding JE, Frieden TR (2004). Local knowledge to enable local action. Am J Prev Med..

[64] Prentice B, Flores G (2007). Local health departments and the challenge of chronic disease: lessons from California. Prev Chronic Dis..

[65] Stamatakis K, Vinson C, Kerner J, Brownson R, Colditz G, Proctor E (2012). Dissemination and implementation research in community and public health settings. *Dissemination and Implementation Research in Health: Translating Science to Practice*.

[66] Green L, Nasser M, Brownson R, Colditz G, Proctor E (2012). Furthering dissemination and implementation research: The need for more attention to external validity. Dissemination and Implementation Research in Health: Translating Science to Practice.

[67] Stamatakis KA, McQueen A, Filler C (2012). Measurement properties of a novel survey to assess stages of organizational readiness for evidence-based interventions in community chronic disease prevention settings. Implementation science : IS.

[68] Stamatakis KA, Ferreira Hino AA, Allen P (2017). Results from a psychometric assessment of a new tool for measuring evidence-based decision making in public health organizations. Eval Program Plann..

[69] Reis R, Duggan K, Allen P, Stamatakis K, Erwin P, Brownson R. Developing a tool to assess administrative evidence-based practices in local health departments. *Frontiers in PHSSR.* 2014;3(3).

[70] Reis RS, Duggan K, Allen P, Stamatakis KA, Erwin PC, Brownson RC (2014). Developing a Tool to Assess Administrative Evidence-Based Practices in Local Health Departments. American journal of public health.

[71] Duckworth AL, Quinn PD (2009). Development and validation of the short grit scale (grit-s). Journal of personality assessment.

[72] Patterson DA, Linn, B., Dulmus, C.N. Are grittier front-line therapists more likely to implement evidence based interventions? Community Academic Partnership on Addiction: Washington University in St. Louis; 2016. 10.1007/s10597-018-0305-1 30083831

[73] Jobe JB, Mingay DJ (1989). Cognitive research improves questionnaires. American journal of public health..

[74] Landis JR, Koch GG. The measurement of observer agreement for categorical data. Biometrics. 1977;33. 843571

[75] Maclure M, Willett WC (1987). Misinterpretation and misuse of the kappa statistic. Am J Epidemiol..

[76] The Guide to Community Preventive Services. Atlanta GA: Centers for Disease Prevention and Control,http://www.thecommunityguide.org/index.html.

[77] US Preventive Services Task Force. Guide to Clinical Preventive Services. 2014; 4th:https://www.uspreventiveservicestaskforce.org/. Accessed February 20, 2017 2017.

[78] Strauss A (1987). *Qualitative Analysis for Social Scientists*.

[79] Strauss A, Corbin J (1990). *Basics of Qualitative Research: Grounded Theory Procedures and Techniques*.

[80] Hruschka DJ, Schwartz D, St. John DC, Picone-Decaro E, Jenkins RA, Carey JW (2004). Reliability in Coding Open-Ended Data: Lessons Learned from HIV Behavioral Research. Field Methods..

[81] Saldana J (2016). *The Coding Manual for Qualitative Researchers*.

[82] Hemming K, Girling A. The efficiency of stepped wedge vs. cluster randomized trials: Stepped wedge studies do not always require a smaller sample size. *J Clin Epidemiol.* 2013;66. 10.1016/j.jclinepi.2013.07.007 24035495

[83] Mdege ND, Man MS, Brown CA Tn, Torgerson DJ. Systematic review of stepped wedge cluster randomized trials shows that design is particularly used to evaluate interventions during routine implementation. *J Clin Epidemiol.* 2011;64. 10.1016/j.jclinepi.2010.12.003 21411284

[84] Robin N, Leep CJ (2017). NACCHO's National Profile of Local Health Departments Study: Looking at Trends in Local Public Health Departments. Journal of Public Health Management and Practice..

[85] Brownson RC, Ballew P, Brown KL (2007). The effect of disseminating evidence-based interventions that promote physical activity to health departments. American journal of public health..

[86] Dobbins M, Hanna SE, Ciliska D (2009). A randomized controlled trial evaluating the impact of knowledge translation and exchange strategies. Implementation science : IS..

[87] Gulliford MC, Adams G, Ukoumunne OC, Latinovic R, Chinn S, Campbell MJ (2005). Intraclass correlation coefficient and outcome prevalence are associated in clustered binary data. J Clin Epidemiol..

[88] Gulliford MC, Ukoumunne OC, Chinn S (1999). Components of variance and intraclass correlations for the design of community-based surveys and intervention studies: data from the Health Survey for England 1994. Am J Epidemiol..

[89] Turner RM, Thompson SG, Spiegelhalter DJ (2005). Prior distributions for the intracluster correlation coefficient, based on multiple previous estimates, and their application in cluster randomized trials. Clinical trials..

[90] Jacobs JA, Duggan K, Erwin P (2014). Capacity building for evidence-based decision making in local health departments: scaling up an effective training approach. Implementation science : IS.

[91] Baio G, Copas A, Ambler G, Hargreaves J, Beard E, Omar RZ (2015). Sample size calculation for a stepped wedge trial. Trials.

[92] Bero LA, Grilli R, Grimshaw JM, Harvey E, Oxman AD, Thomson MA (1998). Closing the gap between research and practice: an overview of systematic reviews of interventions to promote the implementation of research findings. The Cochrane Effective Practice and Organization of Care Review Group. BMJ.

[93] Glasgow RE, Marcus AC, Bull SS, Wilson KM (2004). Disseminating effective cancer screening interventions. Cancer.

[94] Kerner J, Rimer B, Emmons K (2005). Introduction to the special section on dissemination: dissemination research and research dissemination: how can we close the gap?. Health Psychol..

[95] Dreisinger M, Leet TL, Baker EA, Gillespie KN, Haas B, Brownson RC (2008). Improving the public health workforce: evaluation of a training course to enhance evidence-based decision making. J Public Health Manag Pract..

[96] Dobbins M, Robeson P, Ciliska D (2009). A description of a knowledge broker role implemented as part of a randomized controlled trial evaluating three knowledge translation strategies. Implementation science : IS..

[97] Traynor R, DeCorby K, Dobbins M (2014). Knowledge brokering in public health: a tale of two studies. Public Health..

[98] Harris J (2014). *An Introduction to Exponential Random Graph Modeling. Quantitative Applications in the Social Sciences*.

[99] Luke D, Brownson R, Colditz G, Proctor E (2012). Viewing dissemination through a network lens. *Dissemination and Implementation Research in Health: Translating Science to Practice*.

[100] Luke DA, Wald LM, Carothers BJ, Bach LE, Harris JK (2013). Network influences on dissemination of evidence-based guidelines in state tobacco control programs. Health Educ Behav..

[101] Swing States: Missouri: Show Me a Showdown. Retrieved. *The Economist*2008 (August 28).

