# Supplementary material for: Enhancing evidence-based diabetes and chronic disease control among local health departments: a multi-phase dissemination study with a stepped-wedge cluster randomized trial component
Source: Implement Sci. 2017 Oct 18;12:122. doi: 10.1186/s13012-017-0650-4 (PMC5648488; doi:10.1186/s13012-017-0650-4)
Supplement: Supplementary file 1 — Diffusion of Innovation Theory - Stages and Characteristics. (DOCX 38 kb) [file 13012_2017_650_MOESM1_ESM.docx]

**Additional file 1. Diffusion of Innovation Theory – Stages and Characteristics**

| **Dissemination stages** | **Characteristics** |
| --- | --- |
| Innovation | Members from the target audience (LHD employees, in the case of this study) provide critical feedback on the uses of an innovation (EBPPs) |
| Awareness | Defines the actions taken to make target audiences aware of the innovation across sites and settings^1^ |
| Adoption | Decision to use an innovation as the best course of action available^2^ |
| Implementation | Extent to which an innovation is carried out completely and with fidelity^2^ |
| Maintenance | Extent to which an innovation becomes embedded into the normal operation of an organization^3^ |

References

1. Kar SB. Implications of Diffusion Research for Planned Change. *International Journal of Health Education.* 1976;17:192-220.

2. Rogers EM. *Diffusion of Innovations.* Fifth ed. New York: Free Press; 2003.

3. Goodman RM, Tenney M, Smith DW, Steckler A. The adoption process for health curriculum innovations in schools: a case study. *Journal of Health Education.* 1992;23:215-220.
