# Supplementary material for: Enhancing evidence-based diabetes and chronic disease control among local health departments: a multi-phase dissemination study with a stepped-wedge cluster randomized trial component
Source: Implement Sci. 2017 Oct 18;12:122. doi: 10.1186/s13012-017-0650-4 (PMC5648488; doi:10.1186/s13012-017-0650-4)
Supplement: Supplementary file 2 — Examples of Intervention Dissemination Strategies and Description. (DOCX 22 kb) [file 13012_2017_650_MOESM2_ESM.docx]

**Additional file 2. Examples of Intervention Dissemination Strategies and Description**

| **Intervention strategy** | **Description** |
| --- | --- |
| Targeted workshop in evidence-based public health | - Modeled after successful workshops that have been conducted to promote evidence-based decision making.^1,2^ - Seek to enhance participants’ abilities to: understand the role of EBPPs in addressing the diabetes burden; identify diabetes control strategies recommended by evidence-based reviews (e.g., the Community Guide); adapt EBPPs to address health disparities; address organizational barriers to EBPPs; and take action steps to move toward implementation and evaluation of EBPPs. - Incorporate principles of science-based training,^3,4^ including: an informal setting, team training, experiential learning,^3^ and small group activities applicable to real world experiences. - In addition, the study team will sponsor monthly luncheons of workshop attendees to facilitate informal peer knowledge exchange since it has been shown to improve skills.^5^ |
| Knowledge broker | - Provides a link between research and practitioners by developing a mutual understanding of goals and cultures, collaborating with end users to identify problems for which solutions are required, and enhancing access to and use of research evidence in practice and policy.^6^ - Although the public health literature is sparse with evaluations of knowledge broker impact, there is considerable evidence of effectiveness in other fields, particularly from business and agricultural sectors.^7-9^ - The knowledge broker activities include: assistance with needs assessments and strategic planning that incorporate EBPPs; consultation on overcoming barriers to EBPPs; help with grant writing that incorporates information from evidence-based sources; and participation in the dissemination workshops described below. - Interaction will be one-on-one (broker to individuals and teams within the LHD) and include face-to-face contact, telephone, email communication, and a web chat room. |
| Targeted messages | - Involves sending participants a series of emails that include the diabetes control topic and title of a systematic review followed by a link to the full reference, including abstracts similar to the approach successfully used in Canada by Dr. Dobbins.^10^ - Over successive weeks, on a designated day per week determined by the LHD, participants will receive an email:   - Indicating that a systematic review or evaluation related to diabetes or chronic disease control is available.   - It will include a short summary of the research or evaluation, actions that might be taken based on the evidence, and links to evidence-based resources or materials. - The uses and usefulness of targeted messages can be measured in numerous ways by: tracking the number of recipients who open the message link; identifying which content areas are most popular by the frequency of opening links in specific content areas; and at the post-test survey, asking about use of the messages, how they were used, and whether they passed these on to others. |
| Organizational changes | - The study team will work with the LHDs to identify and implement a range of strategies to foster organizational change consistent with Institutional Theory that is part of the study’s conceptual framework (Figure 1). - These strategies will be refined in our planning and based on results from our local-level needs assessment. Examples of strategies that will be employed will seek to: identify ways agency leadership can prioritize use of EBPPs, develop incentives for use of EBPPs, and incorporate EBPPs as a core component of agency practices (e.g., performance reviews, contracts with local partners). |

References

1. Brownson RC, Fielding JE, Maylahn CM. Evidence-based public health: a fundamental concept for public health practice. *Annual review of public health.* 2009;30.

2. Gibbert WS, Keating SM, Jacobs JA, et al. Training the Workforce in Evidence-Based Public Health: An Evaluation of Impact Among US and International Practitioners. *Prev Chronic Dis.* 2013;10:E148.

3. Bryan RL, Kreuter MW, Brownson RC. Integrating Adult Learning Principles Into Training for Public Health Practice. *Health Promot Pract.* Apr 2 2008.

4. Salas E, Cannon-Bowers JA. The science of training: a decade of progress. *Annu Rev Psychol.* 2001;52:471-499.

5. Ramanadhan S, Wiecha JL, Gortmaker SL, Emmons KM, Viswanath K. Informal training in staff networks to support dissemination of health promotion programs. *Am J Health Promot.* Sep-Oct 2010;25(1):12-18.

6. Dobbins M, Robeson P, Ciliska D, et al. A description of a knowledge broker role implemented as part of a randomized controlled trial evaluating three knowledge translation strategies. *Implementation science : IS.* 2009;4:23.

7. Hargadon A. Technology brokering and innovation: linking strategy, practice, and people. *Strateg Leadersh.* 2005;33.

8. Verona G, Prandelli E, Sawhney M. Innovation and virtual environments: towards virtual knowledge brokers. *Organ Stud.* 2006;27.

9. Zook M. The knowledge brokers: venture capitalists, tacit knowledge and regional development. *Int J Urban Reg Res.* 2004;28.

10. Dobbins M, Hanna SE, Ciliska D, et al. A randomized controlled trial evaluating the impact of knowledge translation and exchange strategies. *Implementation science : IS.* 2009;4:61.
